# An Upgraded Smartphone-Based Program for Leisure and Communication of People With Intellectual and Other Disabilities

**DOI:** 10.3389/fpubh.2018.00234

**Published:** 2018-08-28

**Authors:** Giulio E. Lancioni, Nirbhay N. Singh, Mark F. O'Reilly, Jeff Sigafoos, Gloria Alberti, Viviana Perilli, Valeria Chiariello, Serafino Buono

**Affiliations:** ^1^University of Bari, Bari, Italy; ^2^Medical College of Georgia, Augusta University, Augusta, GA, United States; ^3^University of Texas at Austin, Austin, TX, United States; ^4^Victoria University of Wellington, Wellington, New Zealand; ^5^Lega F. D'Oro Research Center, Osimo, Italy; ^6^Oasi Research Institute - IRCCS, Troina, Italy

**Keywords:** leisure, communication, technology, smartphone, intellectual disability, visual impairment

## Abstract

**Background:** People with intellectual disability and sensory or sensory-motor impairments may display serious problems in managing functional daily activities as well as leisure activities and communication with distant partners.

**Aim:** The study assessed an upgraded smartphone-based program to foster independent leisure and communication activity of eight participants with mild to moderate intellectual disability, sensory or sensory-motor impairments, and limited speech skills.

**Method:** The upgraded program was based on the use of (a) a Samsung Galaxy A3 smartphone with Android 6.0 Operating System, near-field communication, music and video player functions, and Macrodroid application, and (b) special radio frequency-code labels. Participants requested leisure and communication activities by placing mini objects or pictures representing those activities and containing frequency-code labels on the smartphone. The smartphone, via the Macrodroid application, read the labels (i.e., discriminated the participants' requests) and provided the participants with the activities requested.

**Results:** During the baseline (i.e., in the absence of the program), the participants failed to request/access leisure and communication activities independently. During the post-intervention phase of the study (i.e., using the program), they succeeded in requesting/accessing those activities independently and spent about 70–90% of their session time busy with those activities.

**Conclusion:** The upgraded smartphone-based program may be highly functional for people like the participants of this study.

## Introduction

People with intellectual disability and sensory or sensory-motor impairments may have serious problems in independently starting and carrying out functional daily activities (e.g., multistep activities concerning domestic and vocational areas such as preparing food and resetting a room) (1–6). The same people may display similarly serious problems in independently managing leisure activities and communication with distant partners (7–11). Regarding functional daily activities, their problems seem to be due to failure in identifying the time when those activities are to be performed and remembering the material and steps involved in the activities (3, 5, 12). Regarding leisure activities and communication with distant partners, their problems seem to be due to failure in (a) finding/locating or effectively using computer systems or other instruments available to access and enjoy those activities (11, 13–15), or (b) using telephone devices and other communication means to get in touch with those partners (16–18).

The damaging consequences of this situation, the general consensus on the need to tackle such a situation, and the increasing emphasis on the role of assistive technology in education, rehabilitation and care contexts have led to the development of a variety of technology-aided intervention programs to reduce the impact of those problems (2, 11, 19–22). To support people's leisure and communication activities, for example, two smartphone-based programs were recently reported (15, 23).

The first smartphone-based program (15) was assessed with five participants with intellectual disability and blindness who possessed easily discriminable speech (i.e., speech skills that were suitable to trigger the S-voice of the smartphone). The smartphone was a Samsung Galaxy A3 with Android 5.1 Operating System, which included standard functions such as S-voice, Internet connection, contacts unit, and media player. It was fitted with a variety of leisure activity files and with catalogs of communication partners and their telephone numbers. The participants would operate the smartphone through the use of specific verbal utterances that triggered the smartphone's S-voice. These utterances enabled them to independently (a) open the leisure activity files and so access the related activities and (b) place telephone calls to the communication partners.

The second program (23) was assessed with five participants with intellectual disability and visual impairment whose speech was inadequate to operate a smartphone (i.e., to trigger its S-voice reliably). To overcome this problem, the program was based on the use of two smartphones such as those employed in the previous study. To make requests for leisure or communication activities, the participants relied on mini objects or pictures fitted with radio frequency-code labels. In practice, the participants placed the objects or pictures on one of the two smartphones. This smartphone decoded the labels attached to the objects or pictures and verbalized the activities they represented. The verbal utterances (a) activated the S-voice of the second smartphone and (b) opened the corresponding leisure activity files or telephone contacts that this smartphone contained, hence allowing the participants to access the leisure and communication activities they had requested.

The results of the aforementioned programs were considered very promising given that the participants learned to operate the technology available and reached high levels of independent leisure and communication engagement. In addition to being effective in helping participants with leisure and communication activities, the smartphone-based programs seemed also relatively practical compared to previous computer-aided programs available in the area (10, 14). Indeed, the smartphone-based programs were simpler and more accessible and affordable than those other programs. Notwithstanding the positive results and potential advantages, smartphone-based programs require additional research attention before one can recommend them for use in daily contexts. In fact, each smartphone-based program was evaluated with only a small number of participants. Moreover, the use of two smartphones for the second program (i.e., to bypass the participants' speech problems), albeit effective, was viewed as a transitory solution waiting to be upgraded. This study was aimed at upgrading the second smartphone-based program (enabling a single smartphone to read and respond to participants' nonverbal requests) and assessing it with eight new participants who presented with intellectual disability, sensory or sensory-motor impairments, and poor speech (24, 25).

## Methods

### Participants

The eight participants recruited for the study (i.e., six men and two women) attended rehabilitation and care centers and represented a convenience sample (26). Table 1 lists them by pseudonyms, and reports their chronological ages, sensory impairments, and Vineland age-equivalent scores for receptive communication (27, 28). The participants' chronological ages ranged from 35 to 58 years. Two participants were diagnosed with total blindness or minimal residual vision (Paul and Darren). The other participants were diagnosed with severe to mild visual impairment (Neil, Peter, Amy, Nancy, and Mat) or no visual impairment (Dan). Two participants were reported to have mild hearing loss, which was corrected through hearing aids (Dan and Paul). In three cases, motor impairments were also present, which precluded or hindered ambulation (Neil, Amy, and Darren).

**Table 1:** Participants' pseudonyms, chronological ages, sensory impairments, and Vineland age-equivalent scores for receptive communication.

**Participants (pseudonyms)**	**Chronological ages (years)**	**Sensory impairments**	**Vineland age-equivalent scores^a^, ^b^**
Neil	58	Mild visual impairment	6;2
Peter	35	Mild/moderate visual impairment	5;1
Amy	42	Severe visual impairment	5;1
Dan	36	Mild hearing loss corrected with hearing aids	3;8
Nancy	43	Mild/moderate visual impairment	5;6
Darren	45	Minimal residual vision	4;3
Paul	36	Total blindness and mild hearing loss corrected with hearing aids	6;6
Mat	37	Moderate/severe visual impairment	4;8

a Vineland age-equivalent scores are reported in “years” (numbers before the semicolon) and “months” (numbers after the semicolon).

b *The age-equivalent scores are based on the Italian standardization of the Vineland scales (19)*.

The participants were included in (considered suitable for) the study based on the following criteria. First, they (a) were functioning in the mild/moderate or moderate intellectual disability range (i.e., as indicated in the psychological records of the rehabilitation and care centers that they attended) and (b) had Vineland age-equivalent scores for receptive communication between nearly 4 years and 6 and a half years (see Table 1). Second, all participants possessed basic speech expressions, which could be understood by staff or relatives, but were inadequate in terms of articulation and clarity to activate the S-voice of the smartphone. Third, the participants enjoyed leisure activities (e.g., listening to music and comedy sketches, watching sport events, and playing guessing games) and telephone calls with family and staff members. However, they needed assistance to access those activities and calls (e.g., they needed somebody to put on the music and set up a call for them). Fourth, staff working with the participants had indicated that they viewed the use of a smartphone-based program, such as that set up for this study, highly valuable for the participants and practical and affordable for the context.

The participants seemed eager to use the program (which had been demonstrated to them). While this eagerness was perceived as consent to the study, their stated inability to deal with a consent form led their legal representatives to step in and provide written informed consent for them. The study complied with the 1964 Helsinki declaration and its later amendments and was approved by a relevant Ethics Committee.

### Setting, sessions, and data recording

The study was carried out in the centers that the participants attended (i.e., in tranquil rooms of those centers). As in previous studies in the area (15, 23), the sessions were implemented for each participant individually, and lasted 15 min or until any activity (e.g., making a telephone call or watching a sport video) requested before the 15-min limit had ended. Sessions were typically carried out two times per day, 5 days a week. Data recording focused on: (a) the leisure and communication activities that the participant managed to request/access independently within the sessions, and (b) the engagement time for each of those activities (i.e., the amount of time elapsing from the request to the end of the activity). Interrater agreement was measured in more than 20% of the participants' sessions, during which a research assistant and a reliability observer were involved in collecting the data. Agreement, which implied that the two data collectors reported identical activities and engagement time periods varying less than 30 s, occurred in more than 90% of those sessions.

### Mini objects, pictures, and activities

Mini objects and pictures, with sizes that did not exceed 7 cm, were used during the intervention and post-intervention phases of the study for Amy, Darren, and Paul, and for Neil, Peter, Dan, Nancy, and Mat, respectively. The mini objects and pictures represented leisure and communication activities, which (a) were considered to be preferred for the participants, (i.e., based on inquiries among staff and participants) and (b) were available for the participants' independent request and subsequent engagement (i.e., via the smartphone-based program). The leisure activities were mainly concerned with (a) listening to singers/songs, local stories, natural science pieces, geography and travel descriptions, comics, food recipes, and quiz games, or (b) watching videos of comedy sketches, and sport events. Videos were available only for Neil and Dan and were shown on their smartphone screen. The communication activities consisted of (i.e., having telephone calls with preferred family or staff members).

The participants were already capable of discriminating/recognizing the mini objects or pictures available for their activity requests. As in the Lancioni et al.'s study (23), (a) the mini objects and pictures were in a container and on a panel, respectively, before the participant, and (b) 12 to 16 mini objects or pictures were present during each session of the post-intervention phase (with about four of those objects/pictures concerning communication activities and the rest concerning leisure activities). Some of the objects and pictures changed over different sessions so as to rotate the 22 or more leisure activities and the six to nine communication partners available to the participants.

### Technology and requests

The technology involved a Samsung Galaxy A3 smartphone device with Android 6.0 Operating System and special radio frequency-code labels (23, 29). The smartphone was equipped with near-field communication, music and video player functions, and Macrodroid application. The smartphone was, moreover, supplied with audio or audiovisual files concerning the leisure activities and telephone partners for communication activities. The frequency-code labels were fixed to the aforementioned mini objects and pictures, which (a) represented the leisure activities available and the partners with whom the participants could talk on the telephone, and (b) were used by the participants to request leisure and communication activities. The Macrodroid application was used to recognize the specific frequency-code labels fixed to the mini objects or pictures and enable such labels to (a) activate telephone calls to the matching communication partners or (b) open the files of the matching leisure activities. Participants could use stereo headphones with an in-line microphone or have the speakerphone turn on automatically during the telephone calls.

In practice, each request consisted of the participant independently selecting a mini object or a picture from the container or panel in front of him or her and placing it onto the back of the smartphone which was next to the container or panel. The Macrodroid application ensured, as explained above, that the participant's request was followed by the matching consequence (i.e., the start of the corresponding leisure activity or telephone call). The same application served also to produce a verbal announcement/reminder during the sessions (e.g., “You can continue with your requests”). No limits were established for the duration of telephone calls, while a limit of about 2 min was set for the leisure activities. These activities, however, would be automatically interrupted before that limit if the participant proceeded with a new request [i.e., as in the Lancioni et al.'s study (23)].

### Experimental conditions and data analysis

The study was conducted in accordance with a non-concurrent multiple baseline design across participants (30). The participants' baseline phase, which varied in numbers of sessions available (i.e., in line with the design), was followed by the intervention and post-intervention phases. Research assistants with experience in the implementation of technology-aided programs with persons with disabilities (and interacting with one another during the study) were in charge of the sessions and recorded the data. The baseline and post-intervention data for independently requested/accessed leisure and communication activities were graphed as means per session over blocks of sessions. A nonparametric statistical test (i.e., Kolmogorov-Smirnov) would be used to assess the differences between phases if overlaps between the two data sets existed (31, 32).

#### Baseline

Each of the three to six baseline sessions available started with the research assistant (a) inviting the participant to use the smartphone on hand (which responded to specific verbal utterances and conventional touch inputs) and (b) indicating activities accessible through it (e.g., listening to singers/songs and making telephone calls). If the participant did not manage to use the smartphone for over 5 min (i.e., as predictable, in light of his or her alleged lack of response skills), the research assistant activated a song, comic sketch, or telephone call for him or her to limit frustration.

#### Intervention

During the 10–14 intervention sessions, the participant had a container with mini objects or a panel with pictures before him or her and the smartphone next to the container or panel. The participant was to practice requesting and accessing leisure and communication activities, by placing the related mini objects or pictures onto the smartphone's back. During the initial sessions, the research assistant helped the participant to make requests through the use of verbal and physical guidance [i.e., as in the Lancioni et al.'s study (23)]. The participant had only seven or eight mini objects or pictures for making the requests (23). During the following sessions, the research assistant's guidance was gradually faded out and the number of mini objects or pictures available was increased. The phase would end with 12 to 16 mini objects or pictures available from the start of the session [see Lancioni et al. (23)]. Those utilized during the session were not replaced.

#### Post-intervention

During the 97–118 post-intervention sessions, conditions matched those in use at the end of the intervention phase. Research assistant's guidance was not available during the sessions. Yet, prior to the sessions the participants were reminded of the activities available and of how they could be requested and accessed.

## Results

**Figure 1:**
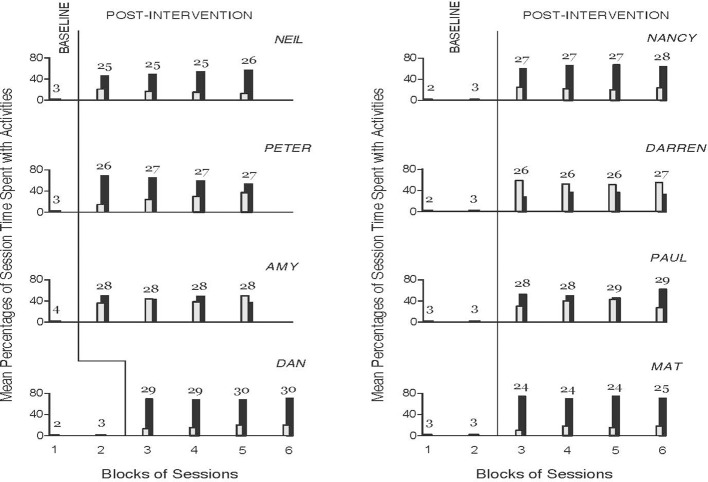
The eight panels provide a graphic view of the data of the eight participants during the baseline and the post-intervention phases. The black and gray bars indicate mean percentages of session time spent with independently accessed leisure and communication activities over blocks of sessions, respectively. The number of sessions included in each block (black- and gray-bar combination) is indicated by the numeral above it.

The eight panels of Figure 1 provide a graphic view of the data of the eight participants during the baseline and the post-intervention phases. The figure does not incorporate the data for the 10–14 intervention sessions, which were focused on introducing the participants to the technology and teaching them to use it to request and access the activities independently. The black and gray bars indicate mean percentages of session time spent with leisure and communication activities requested and accessed independently over blocks of sessions, respectively.

During the baseline sessions, the participants failed to request and access leisure or communication activities through the smartphone on hand, which responded to specific verbal utterances and conventional touch inputs. Therefore, the mean percentages of session time that they spent with independently accessed leisure and communication activities were zero. During the post-intervention sessions (i.e., subsequent to the 10–14 intervention sessions), the participants managed to independently operate the upgraded smartphone technology through the mini objects and pictures and thus succeeded in requesting and accessing leisure and communication activities with regularity. Their overall mean percentages of session time spent with leisure activities requested and accessed independently varied between about 30 (Darren) and 70 (Mat). Their overall mean percentages of session time spent with communication activities requested and accessed independently varied between about 15 (Mat) and over 50 (Darren). The mean cumulative percentages of session time that they spent engaging with the two types of activities were between about 70 and 90. Six participants (Neil, Peter, Dan, Nancy, Paul, and Mat) spent higher percentages of session time with leisure activities. The other two participants (Amy and Darren) spent roughly equivalent or higher percentages of session time with communication activities. Given the zero data values of the baseline sessions and the high cumulative data values of the post-intervention sessions (i.e., given the absence of overlap between the two data sets), no statistical test was deemed necessary to confirm the significance of the differences between phases (31).

## Discussion

The results indicate that the eight participants of this study, who presented with intellectual disability and sensory or sensory-motor impairments and poor verbal skills, successfully used the upgraded smartphone-based program to independently request and access leisure and communication activities. The upgraded program involved a single smartphone, which relied on the use of the Macrodroid application to ensure that the participants' nonverbal requests were discriminated and responded to reliably. On the basis of the results, a few considerations may be put forward.

First, the present study constitutes a clear example of systematic research replication (24) in an area of great practical interest. While this study relied on a new, upgraded technology solution (allowing the use of a single smartphone), the participants were required to produce the same request responses as in the previous Lancioni et al.'s study (23), when the program involved two smartphones. The positive data obtained in the present study strengthen preliminary findings (15, 23) and underline that a smartphone-based program may be a suitable solution for helping people like the participants of this study to manage leisure and communication activities independently (23, 33).

Second, the participants' successful performance during the post-intervention phase of the present study seems to underline two aspects. One aspect concerns their ability to work with the program accurately and without difficulty. The other aspect concerns their apparent interest in engaging in the leisure and communication activities available. The former aspect may underscore the fact that the technology was easy to handle (i.e., required simple responses) and thus was friendly to the participants (33). The latter aspect may emphasize that the participants enjoyed the activities [i.e., found them motivating (34–36)]. Such emphasis is hardly surprising given that the leisure activities and communication partners included in the program were considered preferred for the participants (37–40). While this analysis of the participants' performance appears easily sustainable, the study would have benefited from an investigation of the participants' satisfaction with the program (41). Direct evidence of satisfaction would have added a new dimension to the findings [i.e., it would have suggested that the program has the requisites to improve the participants' positive involvement and quality of life (42–45)].

Third, an upgraded technology solution that involves the use of a single smartphone may be considered more attractive, workable, and acceptable within daily contexts and thus have an increased chance of being adopted in those contexts. In connection with this point, it should be mentioned that the final decision for or against adoption is likely to depend partly on the practicality and effectiveness of the program and partly on staff opinion about (readiness for) the program (46–48). One main limitation of this study is the absence of a social validation of the program, that is, the absence of an assessment of the program based on interviews of staff personnel working in the area. Such an assessment should have determined whether staff personnel recognized the program's alleged strengths and considered it applicable in daily contexts (49–51).

Fourth, new research initiatives should be directed at (a) addressing the main limitations of this study, which were mentioned above (i.e., by setting up an investigation of participant satisfaction and staff opinion), and (b) extending the assessment of the program with additional participants and also via the involvement of new research groups (24, 52). Confirming the present data with additional participants and via studies carried by different research groups would add great strength to the evidence available, and provide sufficient motivation for recommending the program's use on a wider scale (24, 25, 52).

In conclusion, the upgraded smartphone-based program used in this study was effective in supporting leisure and communication of participants with intellectual disability, sensory or sensory-motor impairments, and poor speech, thus replicating and extending previous findings in the area (15, 23). While these results appear very promising and practically relevant, new research initiatives are needed as acknowledged above. Besides those initiatives, one could also consider new developments in the smartphone-based program aimed at making it more easily/profitably applicable among people with special needs (53, 54).

## Author contributions

GL, NS, MO, and JS were responsible for setting up the study, acquiring/analyzing the data, and writing/editing the manuscript. GA, VP, VC, and SB contributed in acquiring and/or analyzing the data and editing the manuscript.

### Conflict of interest statement

The authors declare that the research was conducted in the absence of any commercial or financial relationships that could be construed as a potential conflict of interest.
